# Ozone as an Immunomodulator—New Therapeutic Possibilities in the Treatment of Immunodeficiencies—A Narrative Review

**DOI:** 10.3390/cimb47121016

**Published:** 2025-12-05

**Authors:** Katarzyna Napiórkowska-Baran, Jozef Slawatycki, Paula Klemenska, Paweł Treichel, Ardem Najarian, Gary Andrew Margossian, Maciej Szota, Maria Plocka-Karpinska, Michał Kułakowski

**Affiliations:** 1 Department of Allergology, Clinical Immunology and Internal Diseases, Collegium Medicum Bydgoszcz, Nicolaus Copernicus University, 87-100 Torun, Poland; maciejszota98@gmail.com; 2 Doctoral School of Medical and Health Sciences, Collegium Medicum Bydgoszcz, Nicolaus Copernicus University, 87-100 Torun, Poland; slawatyckijozef@gmail.com (J.S.); treichel.pawel@gmail.com (P.T.); 3 Jan Biziel University Hospital, 85-168 Bydgoszcz, Poland; klemenska.paula@gmail.com; 4 Hollywood Smile Dental Clinic, 62-700 Turek, Poland; centrumedvital@gmail.com; 5 Hollywood Smile Dental Clinic, 62-800 Kalisz, Poland; 6 Faculty of Medicine, New York Medical College, Valhalla, NY 10595, USA; garymargossian@gmail.com; 7 Academia Copernicana Interdisciplinary Doctoral School, Nicolaus Copernicus University, 87-100 Torun, Poland; maria.plocka@wp.pl; 8 Clinical Department of Orthopedics and Traumatology, Jan Biziel University Hospital, 85-168 Bydgoszcz, Poland; mkulakowski@poczta.fm

**Keywords:** ozone therapy, immunomodulation, oxidative stress, inborn errors of immunity, secondary immunodeficiency, Treg cells, redox homeostasis

## Abstract

**Research Subject**: Primary and secondary immunodeficiencies represent a growing clinical and public health challenge due to increased susceptibility to infections, impaired immune regulation, chronic inflammation, and disturbances in redox homeostasis. The pathophysiology of these disorders involves dysfunction of innate and adaptive immunity, altered cytokine production, oxidative stress, and reduced activity of antioxidant defense mechanisms. In recent years, attention has increasingly focused on the role of oxidative imbalance and chronic inflammation in weakening immune function. Ozone therapy, when used at controlled low doses, induces a hormetic response that triggers adaptive antioxidant pathways, modulates cytokine profiles, and enhances the activity of immune cells. Due to these properties, ozone has emerged as a potential adjunctive therapy aimed at restoring immune homeostasis and improving clinical outcomes in patients with immune disorders. **Aim of Study**: The aim of this review is to discuss the role of oxidative stress and immune dysregulation in the pathogenesis of immunodeficiencies and to provide an updated overview of current evidence regarding the therapeutic potential of ozone therapy. This article summarizes molecular mechanisms, biochemical effects, and clinical findings related to ozone-based interventions, with particular emphasis on cytokine modulation, redox balance, macrophage function, regulatory T cells (Treg), and NK cell activity. **Materials and Methods**: This review is based on scientific data retrieved from PubMed, Scopus, and Google Scholar. Included sources comprise randomized clinical trials, observational studies, meta-analyses, mechanistic studies, and review articles published between 1996 and 2025. Keywords used during the literature search included: “ozone therapy”, “immunomodulation”, “oxidative stress”, “inborn errors of immunity”, “secondary immunodeficiency”, “Treg cells”, “redox homeostasis”. **Results**: Analysis of current studies shows that controlled low-dose ozone (typically 10–40 µg/mL) activates the Nrf2/ARE antioxidant pathway, increases enzymatic defense (SOD, catalase, GPx), and reduces levels of proinflammatory cytokines such as TNF-α, IL-1β, and IL-6. Clinical trials report improved lymphocyte profiles, enhanced macrophage phagocytic function, increased Treg activity, and reinforced NK cell responses. Patients receiving ozone therapy demonstrate reductions in inflammatory markers (CRP, IL-6, D-dimer), improved redox balance, decreased infection frequency, and better overall immune performance. The therapy is generally well tolerated when administered within established safety guidelines. **Conclusions**: Available evidence indicates that ozone therapy may serve as a valuable adjunct in the management of immunodeficiencies by modulating immune responses, reducing oxidative stress, and restoring homeostatic balance. Although current clinical outcomes are promising, further multicenter randomized trials are needed to standardize dosing protocols, assess long-term effectiveness, and confirm safety.

## 1. Introduction

The immunomodulatory effects of ozone have been recognized for many years, and they have recently gained increasing significance in the context of translational medicine, supporting the treatment of individuals with immunodeficiencies. The rising incidence of immune dysfunctions—such as inborn errors of immunity (IEIs) and secondary immunodeficiencies (SID)—highlights the need to identify effective and safe adjunctive therapies [1,2]. Ozone, a non-radioactive gas with strong oxidative properties, exhibits immunomodulatory activity, making it a potential therapeutic tool in this field [3].

In recent years, ozone therapy has made remarkable progress. According to the Madrid Declaration, ozone therapy is defined as a “medical procedure” applied in human medicine, dentistry, and veterinary medicine (https://isco3.org/madrid-declaration-on-ozone-therapy-3rd-edition-isco3/, accessed on 29 November 2025). Evidence of the growing recognition of this therapeutic method was the Round Table Meeting at the European Parliament held on 14 February 2024, titled “New Horizons for Healthcare in Europe: The Medical Ozone Therapy and Its Multiple Benefits.” The event was attended by five European ozone therapy physicians, including two members of AEPROMO and ISCO3. This meeting represented a significant step forward, marking ozone therapy as an officially recognized complementary treatment (https://aepromo.org/en/ozone-therapy-was-heard-loud-and-clear-in-the-european-parliament/, accessed on 29 November 2025).

It is now only a matter of time before this therapy gains widespread acceptance and integration into everyday clinical practice.

This article aims to present current data on the immunomodulatory effects of ozone and to assess its potential therapeutic impact in patients with immunodeficiencies. The main research questions are: (1) How does ozone influence innate and adaptive immunity in individuals with IEIs and SID? and (2) Can ozone therapy be used to modulate immunological parameters and reduce the frequency of infections in this patient group?

The article draws on data from peer-reviewed clinical and preclinical studies, as well as systematic reviews—including recent publications—addressing the biochemical and cellular mechanisms of ozone action. Particular attention is given to the hormetic effect, regulation of inflammatory responses **via** the Nrf2/ARE and NF-κB pathways, modulation of pro- and anti-inflammatory cytokines, and the interaction of ozone with the antioxidant defense system.

The discussion also encompasses safety aspects, appropriate dose selection, and routes of administration, as well as therapeutic protocol standards and the potential synergy of ozone therapy with immunonutrition and trace elements.

Existing studies suggest that ozone, in small and precisely controlled doses, can induce adaptive processes at the cellular level, enhance antioxidant enzyme activity, and regulate the cytokine profile [4]. It has been demonstrated that ozone exposure leads to a decrease in pro-inflammatory cytokine activity, while increasing anti-inflammatory cytokine levels, thereby promoting the restoration of homeostasis and improving the body’s immunological defense [5]. However, it should be emphasized that there is still an insufficient number of studies evaluating the long-term effects of ozone therapy, its interactions with other treatment modalities, and the potential variability in individual patient responses. Therefore, it is crucial to conduct further well-designed clinical trials and to establish international standards for ozone dosing and clinical application criteria.

## 2. Immunodeficiencies

### 2.1. Immunodeficiencies—Challenges of Modern Medicine

Immunodeficiencies represent a significant challenge for modern medicine [6]. By definition, immunodeficiencies are pathological conditions associated with dysfunction or insufficiency of the immune system. They are classified into primary immunodeficiencies (PIDs), currently referred to as inborn errors of immunity (IEIs), and secondary (acquired) immunodeficiencies (SIDs) [7].

IEIs are characterized by increased susceptibility to infections, malignancies—mainly of lymphoid origin—and autoimmune diseases. According to the International Union of Immunological Societies (IUIS) classification, IEIs include, among others: combined immunodeficiencies (involving both cellular and humoral immune defects), predominantly antibody deficiencies, other well-defined immunodeficiencies, chromosomal instability syndromes, complement deficiencies, and phagocytic disorders [7,8].

Secondary immunodeficiencies (SIDs) are caused by external factors, including immunosuppressive drugs, malignancies, malnutrition, diabetes, extensive wounds, renal or hepatic failure, infections—particularly of viral etiology (e.g., EBV, HIV, CMV)—autoimmune diseases, or postsplenectomy status [7,9,10].

The treatment of immunodeficiencies remains challenging due to the complexity of immunological mechanisms and the difficulty in precisely identifying the underlying cause. Available therapeutic methods are often expensive, carry a risk of complications, and do not always result in complete restoration of immune function. In many cases, management is primarily symptomatic (e.g., antibiotic therapy) and requires long-term, multidisciplinary patient care [11].

An important aspect of therapeutic management is the use of supportive therapies aimed at restoring immunological homeostasis, which can enhance the effectiveness of causal treatment. These include appropriate supplementation (e.g., vitamin D3, B-group vitamins), lifestyle modifications, probiotic therapy, reduction in oxidative stress, and a properly balanced diet [12,13].

### 2.2. Ozone as a Biologically Active Agent

**Figure 1:**
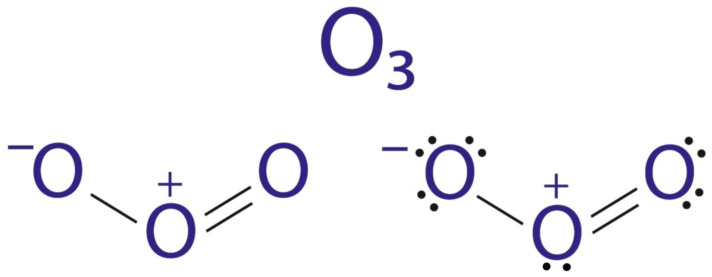
Chemical Structure of Ozone.

Ozone (O_3_) is a triatomic form of oxygen characterized by strong oxidative and disinfectant properties. Under standard conditions, it exists as a bluish gas with a sharp odor, exhibiting high chemical reactivity and significant instability, which manifests in its rapid decomposition into molecular oxygen (O_2_), especially under the influence of high temperature and ultraviolet radiation [14]. For this reason, ozone must be generated immediately before use, typically with ozone generators. Figure 1 shows the chemical structure of ozone.

At low doses, ozone exhibits antimicrobial and anti-inflammatory effects, as well as the ability to improve tissue oxygenation [15]. At high doses, however, ozone is toxic to tissues and therefore requires strict clinical control during its application [16].

Ozone therapy was initially used primarily in topical form, including wound and ulcer disinfection, as well as in dental procedures, which leveraged the strong oxidative properties of ozone [17]. As clinical research developed in the 1930s and 1940s, intravenous ozone administration was explored, leading to the development of autohemotherapy—a procedure involving extracorporeal ozonation of blood followed by its reinfusion.

Modern approaches to ozone therapy regard ozone not only as an antiseptic agent but primarily as a bioregulator capable of modulating key physiological and immunological processes through the stimulation of endogenous antioxidant mechanisms, improving microcirculation, and modulating inflammatory and immune responses. Today, ozone therapy is applied in medicine for the treatment of chronic wounds, infections, and joint diseases [18].

## 3. Mechanisms of Ozone Action Within the Immune System

The effects of ozone on the immune system are based on adaptive mechanisms and the modulation of inflammatory processes. The foundation of these effects lies in hormesis and the regulation of immune mediators, which together enable the strengthening of the immune response. These processes occur at both the molecular and cellular levels and will be discussed in detail in the following subsections.

### 3.1. The Concept of Hormesis and Low-Intensity Oxidative Stress

Depending on the administered dose, ozone can produce either toxic effects (at doses above the recommended range) or beneficial health effects (at low, therapeutically recommended doses). This phenomenon is described as ozone hormesis.

Ozone hormesis involves the induction of mild oxidative stress in the organism through the administration of low ozone concentrations (10–40 µg/mL), which stimulates adaptive defense mechanisms [19,20]. This process activates several molecular pathways, including the Nrf2/ARE pathway, resulting in increased expression of endogenous antioxidant enzymes such as superoxide dismutase (SOD), catalase, and glutathione peroxidase (GPx). These enzymes enhance immune response cells’ resistance to the harmful effects of reactive oxygen species (ROS) and restore redox homeostasis.

The anti-inflammatory activity of ozone likely stems from its ability to regulate the cellular microenvironment by reducing the persistence of oxidative stress [21]. The intracellular concentration of H_2_O_2_ is significantly lower than that of other radicals such as O_2_•^−^, which indicates a well-balanced redox homeostasis within cells [22]. Unlike conventional antioxidants, ozone acts on entire adaptive cellular pathways, influencing both the expression of enzymes and the production of reactive metabolites, while also possessing strong oxidative potential [19,23].

In turn, NF-κB is the main transcription factor involved in the inflammatory response, initiating the expression of pro-inflammatory cytokines such as TNF-α, IL-1β, and IL-6, whose levels increase following ozone exposure. Ozone, as a potent oxidizing agent, can simultaneously activate NF-κB and modulate the Nrf2 pathway, leading to a dynamic balance between pro-inflammatory and antioxidant processes. Consequently, the mutual regulation of the Nrf2/ARE and NF-κB pathways determines the intensity of the inflammatory response and the degree of tissue damage in individuals with impaired immunity exposed to ozone-induced oxidative stress [24].

Both primary and secondary immunodeficiencies are associated with disturbed redox balance and excessive oxidative stress, which contribute to cellular damage and dysregulation of immune responses. Under conditions of reduced activity of antioxidant enzymes such as SOD, catalase, and glutathione peroxidase, accumulation of reactive oxygen species (ROS) occurs, promoting inflammation and apoptosis of immune cells, ultimately impairing immune function. Properly adjusted ozone doses may therefore be highly beneficial in treating patients with primary and secondary immunodeficiencies. Lipid peroxidation and DNA damage are reduced in cells chronically exposed to oxidative stress due to IEIs and SID [19,23].

Ozone-induced hormetic mechanisms limit the progression and chronicity of tissue damage caused by oxidative stress. These adaptations strengthen the immune system and reduce the risk of damage in chronic oxidative conditions by activating detoxifying enzymes, DNA repair mechanisms, and anti-inflammatory pathways [22,23]. This process helps restore defensive mechanisms and enhances resistance to both extracellular and intracellular microorganisms. At the same time, nearly all oxygen within cells is utilized efficiently in metabolic processes [22].

The dose–response curve confirms the hormetic nature of ozone’s action—an adaptive response occurs only at low doses, whereas high doses induce toxicity. Therefore, ozone therapy must be individually tailored and carefully monitored. Exceeding therapeutic ozone concentrations can lead to cytotoxicity and tissue injury, making accurate dosing crucial for minimizing the risk of adverse effects [20,25].

### 3.2. The Effect of Ozone on Innate and Adaptive Immunity

#### 3.2.1. Ozone and Innate Immunity

The regulation of the immune response by ozone is based on a highly complex mechanism involving a wide range of molecular and cellular phenomena. It encompasses both innate and adaptive immunity.

Ozone therapy demonstrates the potential to modulate macrophage functions through two main pathways: by enhancing their phagocytic capacity and by modifying the profile of secreted cytokines. Ozone significantly affects macrophage activity by inducing moderate oxidative stress, which triggers cellular adaptive mechanisms via activation of the Nrf2/ARE signaling pathway. As a result, the expression and activity of key antioxidant enzymes such as superoxide dismutase (SOD), catalase (CAT), and glutathione peroxidase (GPx) increase. This mechanism not only strengthens the cells’ defense capabilities but also enhances their phagocytic functions, thereby improving the efficiency of pathogen elimination. To achieve a beneficial therapeutic effect, it is essential to appropriately harness the phenomenon of ozone hormesis—that is, to apply a dose sufficiently low to stimulate adaptive immune processes while remaining below the threshold that would cause cellular damage.

Studies by Malatesta et al. [23,25] confirmed that exposure of macrophages to controlled doses of ozone enhances their phagocytic capacity, which may be particularly important in immunocompromised patients prone to frequent opportunistic infections. The authors also observed that ozone therapy prevented further decline in circulating monocytes in patients with advanced neurodegenerative diseases, suggesting that ozone may support the regeneration of functional immune cells in conditions associated with chronic oxidative stress.

The effect of ozone, which induces moderate oxidative stress, plays a crucial role in regulating the immune response. It not only activates macrophage adaptive mechanisms via the Nrf2/ARE pathway and enhances the activity of antioxidant enzymes (SOD, CAT, GPx) but also modulates the cytokine secretion profile. This regulation leads to increased production of anti-inflammatory cytokines such as interleukin-10 (IL-10) and transforming growth factor β (TGF-β), thereby limiting excessive pro-inflammatory immune activity and restoring immunological homeostasis.

As reported by Tahmasebi et al. [26], moderate oxidative stress induced by ozone promotes macrophage adaptability, reducing the risk of chronic inflammation. This effect is of particular clinical significance in patients with primary or secondary immunodeficiencies, where persistent inflammation represents a key pathogenic factor. The ability to limit excessive pro-inflammatory macrophage activity while maintaining their phagocytic and pathogen-eliminating capacity allows for an optimal therapeutic outcome and supports the restoration of proper immune function.

Ozone also affects cells involved in the innate immune response, including macrophages, neutrophils, monocytes, dendritic cells, and natural killer (NK) cells. Ozone can restore normal cell counts in conditions of chronic deficiency while maintaining their phagocytic and migratory functions [23,27]. Notably, immune stimulation by ozone does not adversely affect cells responsible for downregulating immune activity, which is crucial for preventing infections and in cases of immune exhaustion.

Another key cell population influenced by ozone is the NK cell—an essential component of antiviral and antitumor immunity. Bocci et al. [28] demonstrated that ozone therapy enhances the capacity of NK cells to eliminate both virus-infected and malignant cells, with the maintenance of a balance between pro-inflammatory and anti-inflammatory cytokines playing a critical role in this process. The ozone-mediated reduction in TNF-α production contributes to strengthening the antiviral and antitumor effects of NK cells. This modulation is particularly relevant in patients with secondary immunodeficiencies, where NK cell function is often impaired.

Furthermore, reports are suggesting the potential for synergistic NK cell activation when ozone therapy is combined with other immunological strategies, such as immunoglobulin administration or cytokine therapy. However, this approach requires confirmation in well-designed, randomized clinical trials to assess its safety and efficacy.

In summary, the effects of ozone therapy on the cytotoxic activity of NK cells in IE-Is are still limited, but available studies suggest a positive effect. In vitro, short-term exposure of human PBMC to low ozone concentrations (1–5 µg/mL) increased the per-centage of CD3–CD16+/CD56+ (NK) cells and their cytotoxicity [29]. These results indicate an enhanced immune response and reduced infectious symptoms after ozone therapy. The importance of NK function in IEIs is further emphasized by a large cohort of CVID patients, in which patients with severe NK lymphopenia had a significantly higher rate of invasive bacterial infections and pneumonia [30]. Although in the same analysis the incidence of cancer did not differ significantly between groups with different NK cell counts, the correlation between NK cell counts and infections suggests that increased NK activity may reduce susceptibility to infections. In summary, direct studies of NK cytotoxicity in patients with PID after ozone therapy do not yet exist, but observations from other models (especially the increase in NK cytotoxicity after ozone exposure and the improvement of immunological and clinical parameters in patients with IgA deficiency after ozone treatment) indicate that ozone may stimulate NK cell function and thus potentially reduce the number of infections. Much probably depends on the balancing effect (ozone reduces chronic TNF-α and inflammation) so as not to reduce it below the level needed for NK activation [29]. However, the current state of knowledge suggests that ozone therapy models cytokines and oxidative stress in a way that leads to a general improvement in immune mechanisms, which remains to be confirmed in clinical studies correlating NK function with the frequency of infections and neoplastic events.

#### 3.2.2. Ozone and Adaptive Immunity

Ozone exerts a significant influence on adaptive immunity especially on the population of regulatory T lymphocytes (Treg). It increases their number and activity, while inducing the expression of FoxP3, a transcription factor crucial for Treg function. This mechanism helps suppress excessive autoimmune reactions and supports the maintenance of immunological homeostasis.

Tahmasebi et al. [26] demonstrated that ozone therapy increases the number of Treg cells and the levels of anti-inflammatory cytokines, such as IL-10 and TGF-β, in patients with multiple sclerosis. As a result, autoimmune activity and chronic inflammation are reduced, particularly in individuals with an impaired balance between effector and regulatory cells.

The influence of ozone on the Treg population may also reduce the risk of cytokine storm and promote the induction of immune tolerance, thereby minimizing potential inflammatory reactions. Additionally, ozone therapy has been shown to affect the expression of specific microRNAs, including miR-17, miR-27, and miR-146a, which play regulatory roles in immune response modulation. This mechanism suggests a broader immunoregulatory spectrum of ozone activity, involving modulation of key immune signaling pathways. As emphasized by the authors [26], further studies are needed to fully elucidate the role of ozone-induced microRNAs in regulating Treg activity.

In summary, ozone therapy—by modulating moderate oxidative stress—enhances innate immunity by improving macrophage phagocytic activity and NK cell function, while regulating the balance between pro- and anti-inflammatory cytokines. In the domain of adaptive immunity, ozone supports the population of regulatory T cells (Treg) by inducing FoxP3 expression and increasing anti-inflammatory cytokines (IL-10, TGF-β), thereby maintaining immune homeostasis and limiting autoimmune responses. These effects make ozone therapy a promising adjunctive tool for modulating immune function in patients with immunodeficiencies or chronic inflammation.

### 3.3. Regulation of Inflammatory Mediators

Ozone can modulate the immune response by influencing inflammatory mediators—both pro-inflammatory cytokines (IL-6, TNF-α) and anti-inflammatory ones (IL-10). The reduction in pro-inflammatory cytokine levels, accompanied by an increase in anti-inflammatory cytokines, leads to a balanced immune response. The suppression of excessive inflammatory mediator production by low doses of ozone has been confirmed in studies by de Sire et al. [31] and Delgado-Roche et al. [32] in patients with primary immunodeficiency (IE) and secondary immunodeficiency (SID).

The use of controlled, low doses of ozone induces a hormetic effect, which prevents the progression of inflammation in both acute and chronic disease states. As demonstrated by de Sire et al. [31], the hormetic effect of ozone allows the immune response to be adjusted to the organism’s current needs. This effect, characterized by the reduction in IL-6 and TNF-α levels and the increase in IL-10 and TGF-β concentrations, contributes to the attenuation of inflammatory activity in rheumatic diseases and promotes the induction of immune tolerance in IEIs and SID [26,27,32]. This modulation is crucial for terminating immune responses during inflammation treatment and improving immune function. Shifting the cytokine balance is particularly important when standard pharmacological anti-inflammatory strategies are ineffective or contraindicated. The mechanism of ozone action in patients with severe inflammatory conditions may minimize the risk of cytokine storm during infections [27].

Ozone may also modulate the secretion of prostaglandins and leukotrienes in the context of immunodeficiency, thereby supporting the restoration of immune balance. By regulating the activity of cyclooxygenase (COX) and 5-lipoxygenase (5-LOX) enzymes, ozone therapy can decrease the production of pro-inflammatory prostaglandins, such as PGE2, and leukotrienes, such as LTB4, whose excess intensifies inflammation and weakens immune responses. As a result, it limits the excessive activation of neutrophils and effector cells, contributing to the reduction in chronic inflammation. At the same time, the modulation of these mediators by ozone helps maintain the balance between the inflammatory response and the body’s defense mechanisms—a factor of particular importance in patients with exhausted or weakened immune systems.

At the molecular level, ozone activates the Nrf2/ARE pathway, increasing the expression of antioxidant enzymes such as SOD, catalase, and GPx, while inhibiting NF-κB activity, which leads to reduced transcription of pro-inflammatory mediators. Moreover, ozone suppresses the NLRP3 inflammasome, resulting in decreased caspase-1 activity and lower IL-1β levels [24,33].

An indirect mechanism of ozone action involves the enhancement of antioxidant processes, which supports tissue regeneration and decreases the concentrations of pro-inflammatory factors such as IL-6 and CRP [31,34]. In clinical practice, this translates into a reduction in perceived pain among patients. Ozone helps maintain cellular redox balance, prevents lipid peroxidation, and reduces cellular damage. Activation of antioxidant enzymes, combined with a reduction in pro-inflammatory factors, leads to the elimination of reactive oxygen species (ROS) and peroxides that play a key role in chronic inflammation [25,32].

Clinical and preclinical studies have demonstrated that ozone therapy reduces the incidence of secondary tissue damage and infections, as well as the risk of complications associated with immunosuppression [35]. Ozone acts preventively against cytokine cascade activation, restoring balance between pro- and antioxidant mechanisms in chronic inflammation. Studies by Malatesta et al. (2024) and de Sire et al. (2022) [25,31] confirmed that ozone activates adaptive cellular mechanisms that decrease inflammatory mediator levels and enhance endogenous antioxidant concentrations. Table 1 shows a summary of the immunomodulatory effects of ozone.

## 4. Clinical Studies

Preliminary data also suggest the potential application of ozone therapy in patients with primary immunodeficiencies. In a randomized clinical trial, Díaz-Luis et al. reported that patients with primary IgA deficiency experienced a significant increase in IgG and IgM levels (IgG at baseline 8.2, after intervention 12.0; IgM at baseline 0.07, after intervention 0.12). After two cycles of ozone therapy, the study group also had improvements in oxidative stress markers and antioxidant enzymes activity compared to placebo group. These results confirm the immunostimulatory potential of ozone and its clinical safety [36].

As emphasized by Szaflarska et al., in the field of primary immunodeficiencies such as CVID and IgA deficiency, there is an urgent need to develop new and effective immunomodulatory therapies. Due to its antioxidant and immunoregulatory properties, ozone therapy may represent a promising alternative therapeutic approach worthy of further clinical investigation [37].

Ozone therapy is increasingly being used as an adjunctive treatment in patients with primary immunodeficiencies—such as IgA deficiency and common variable immunodeficiency (CVID)—as well as in secondary immunodeficiencies, including HIV infection, malignancies, and post-COVID syndrome (so-called long COVID). Studies by Franzini et al. and Martínez et al. [38,39] demonstrated that ozone therapy leads to a significant reduction in inflammatory markers (CRP, IL-6, D-dimer) and improves both redox balance and immune activity. In patients with COVID-19 treated with ozone autohemotherapy, an increase in lymphocyte count and in the CD4/CD8 ratio was observed, confirming its beneficial immunoregulatory effects. Similar outcomes were noted in HIV-infected individuals. Long-term use of ozone as an adjunct to antiretroviral therapy resulted in an increase in CD4 lymphocyte percentages and overall improvement in immune activity, with no reported adverse effects [40].

It is worth emphasizing that, despite etiological differences, IEIs such as CVID or isolated IgA deficiency, as well as infectious and inflammatory diseases (COVID-19, HIV, rheumatoid arthritis, chronic periodontitis), share common immunological elements. Patients with CVID are characterized by hypogammaglobulinemia (low IgG and IgA levels) and recurrent upper respiratory tract infections, as well as an increased risk of lymphatic malignancies and auto-immune disorders. This is accompanied by persistent abnormalities in innate immunity—CVID patients exhibit, among other things, a reduced number of natural killer cells and the presence of pro-inflammatory macrophage monocytes secreting cytokines such as TNF-α [36]. Similarly, chronic inflammatory states are associated with excessive production of TNF-α, IL-6, and IL-1β, which occurs in severe forms of COVID-19, as well as in RA and chronic per-iodontitis. Ozone therapy, in turn, demonstrates universal immunomodulatory effects—initiating an antioxidant response by activating the Keap1/Nrf2/ARE pathway and reducing levels of proinflammatory cytokines (TNF-α, IL-6, IL-1β) [39]. In light of these findings, it can be concluded that, although CVID and IgA deficiency are primary immune disorders with a different cause than HIV or COVID-19, they are accompanied by similar patterns of chronic immune stimulation and oxidative stress. This similarity justifies cautious extrapolation of ozone’s effects between these conditions, especially since the goals of therapy (including reducing excessive cytokines and supporting antioxidant mechanisms) are convergent in these pathophysiological conditions [36,39].

### 4.1. Local and Systemic Ozone Therapy

Ozone therapy—both local and systemic—is used in the treatment of patients with primary and secondary immunodeficiencies. Both ozone autohemotherapy and ozonated water therapy lead to improvement in immunological parameters and reduction in infection frequency. Studies have shown that systemic administration of ozone in COVID-19 patients shortened hospitalization time, reduced proinflammatory cytokine levels (IL-2, IL-6, IL-8, TNF-α), and simultaneously increased concentrations of the anti-inflammatory cytokine IL-10 [41].

Ozone autohemotherapy, which involves the introduction of ozone into the bloodstream, has an immunomodulatory effect on the entire body. Ozonated water, administered rectally or topically to mucous membranes, has local anti-inflammatory effects, adapting its action to the site-specific needs [42].

Systemic ozone therapy can be considered to improve biochemical parameters, reduce CRP levels, and lower the incidence of infectious complications in patients with immune dysfunction [41]. The effectiveness of local methods has also been confirmed in animal studies, where rectal administration of ozonated water activated the SIRT1–Nrf2/HO-1 pathway, resulting in improved intestinal barrier integrity, modulation of the microbiome, and suppression of inflammatory responses in infectious immune disorders [43].

Autohemotherapy activates key adaptive pathways, including the Nrf2/ARE pathway, which increases the expression of antioxidant enzymes such as superoxide dismutase, catalase, and glutathione peroxidase. As a result, immune control mechanisms improve, and secondary oxidative stress is reduced [41]. In patients with chronic inflammatory conditions, such as rheumatoid arthritis, this therapy improves both subjective well-being and biochemical parameters [41,44].

Moreover, normalization of the cytokine profile has been observed, with decreased levels of proinflammatory cytokines (IL-2, IL-6, and IL-8) and increased IL-10 expression, leading to modulation of the immune response and a reduction in the risk of cytokine storm [41].

Rectal administration of ozonated water activates the SIRT1–Nrf2/HO-1 pathway, promoting epithelial regeneration, reducing inflammatory infiltration (as indicated by CD11b and F4/80), and restoring intestinal barrier integrity (Su et al., 2025) [43]. Concurrently, favorable changes in the microbiome have been observed, including increased abundance of Lactobacillaceae and Adlercreutzia species [43].

Enhancement of innate immunity contributes to a reduced risk of secondary infections.

Topical application of ozonated water is also effective in treating fungal and bacterial infections of the oral cavity, as well as in preventing postoperative complications, supporting wound healing, and reducing infection rates [44]. Systemic ozone therapy via autohemotherapy has a beneficial effect on both innate immunity (by increasing phagocyte activity) and adaptive immunity (by modulating T lymphocyte function) [45]. An increase in regulatory T cell numbers and a decrease in proinflammatory cell activity have been observed, suggesting a promising strategy for reducing autoimmune responses. Additionally, animal studies have shown that ozonated water increased systemic antioxidant activity after microbiota transplantation from COVID-19 patients [43].

Randomized clinical trials (RCTs) confirm the efficacy of ozone therapy in improving patients’ clinical condition—reducing pain, enhancing physiological function, and improving quality of life. Therapeutic effects may persist for up to six months after treatment completion, with a high safety profile maintained [46,47]. According to recommendations, ozone infusion concentrations of 10–40 µg/mL are used [19]. Ozone, acting via a hormetic effect, activates adaptive cellular mechanisms, making it a safe therapeutic tool in chronic conditions such as immunodeficiencies. Similarly to balneotherapy, the moderate oxidative stress induced by ozone helps reduce chronic inflammation [48].

However, further development of ozone therapy requires standardization of key parameters—dosage, volume, administration time, and clinical indications. Combining this method with immunonutrition and conventional pharmacological treatment may reduce the need for immunosuppression, thereby improving patients’ quality of life [31,46].

### 4.2. Synergy with Other Therapies

It is worth noting that while ozone at low concentrations exhibits a multi-level, modulatory effect on the functioning of immune system cells, at high concentrations it can act toxically, causing damage to pulmonary epithelial cells, stimulating inflammatory responses, disrupting epithelial barrier integrity, and contributing to the development of chronic diseases [49]. Under controlled conditions, however, with ozone doses in the range of 10–40 µg/mL, a phenomenon of hormetic oxidative stress is observed. This effect, through modulation of the endogenous antioxidant system, leads to cellular adaptation and improved resistance to stress factors without inducing adverse effects [21].

Viebahn-Hänsler et al. [50] demonstrated that systemic ozone therapy, when administered according to appropriate protocols, is a safe and side-effect-free method. Moreover, combining ozone therapy with immunonutrition, which involves key micronutrients such as zinc, selenium, and vitamin C, may further enhance therapeutic efficacy in patients with immune dysfunction. As indicated by Iddir et al. these micronutrients support the activity of antioxidant pathways and exhibit synergistic effects with ozone in minimizing inflammatory processes and oxidative stress [51].

Providing adequate nutritional components in combination with ozone therapy helps reduce chronic oxidative stress and improve immune system function by stabilizing the populations of effector immune cells. The synergistic action of ozone and micronutrients leads to a reduction in inflammation intensity, regulation of the balance between proinflammatory and anti-inflammatory cells, and improvement in the proliferation, differentiation, activation, and phagocytic and cytotoxic functions of immune cells [51]. The summary of ozone applications in medicine is presented in Table 2.

## 5. Safety and Regulatory Aspects

The therapeutic range of ozone activity, referred to as the “safety window,” constitutes a key element in the rational and safe clinical application of ozone therapy. It encompasses the concentration range within which ozone induces desirable biological effects while avoiding toxic consequences resulting from excessive oxidative stress. According to international guidelines from the World Federation of Ozone Therapy (WFOT) and the International Scientific Committee of Ozone Therapy (ISCO3), the safe therapeutic range of ozone includes concentrations of 10–40 µg/mL for systemic applications and 60–100 µg/mL for local procedures with antibacterial and regenerative effects [19]. Within this range, ozone exhibits immunomodulatory, anti-inflammatory, and antioxidant properties, whereas excessive concentrations may lead to unfavorable oxidative reactions, cellular membrane damage, lipid peroxidation, and redox imbalance.

Although ozone therapy is considered a procedure with a high safety profile, the literature describes occasional adverse effects such as transient headaches, fatigue, inflammatory reactions at the injection site, cough after autohemotherapy, or mild allergic reactions [48]. Serious complications are infrequent and most often result from technical errors, such as improper ozone concentration, use of contaminated gas mixtures, or incorrect administration routes (e.g., extravascular intravenous injection). Absolute contraindications include glucose-6-phosphate dehydrogenase deficiency, severe hyperthyroidism, unstable cardiovascular diseases, thrombocytopenia, and pregnancy. Proper patient qualification, therapy supervision, adherence to aseptic technique, and accurate parameter selection are essential for ensuring procedural safety [52].

WFOT and ISCO3 guidelines clearly emphasize the need for standardization and harmonization of therapeutic methods to ensure comparability of clinical outcomes. This primarily concerns the validation of parameters such as ozone concentration, gas mixture volume, treatment frequency, and administration route, which should be defined based on scientific evidence and monitored in clinical registries. The lack of unified protocols across countries poses a significant barrier to assessing the efficacy and safety of ozone therapy, hindering its full integration into evidence-based medicine. Therefore, prospective, multicenter clinical studies with high methodological rigor are essential to validate procedures and establish global therapeutic standards. The implementation of international regulations in this area is a necessary condition for the further development and safe dissemination of ozone therapy in clinical practice [53,54].

## 6. Future Research Directions

Future research on ozone therapy should focus on an in-depth evaluation of its clinical efficacy, safety, and molecular mechanisms of action, particularly in the context of immunological disorders such as inborn errors of immunity (IEIs) and secondary immunodeficiency disorders (SID). Despite the growing number of preclinical and clinical observations indicating the immunomodulatory properties of ozone, there remains a lack of prospective, randomized, placebo-controlled trials that can conclusively confirm its efficacy and define optimal therapeutic protocols for these conditions [55]. It is particularly necessary to determine to what extent various dosing regimens and routes of administration (autohemotherapy, insufflation, local application) modulate the immune, redox, and metabolic responses of the organism [56].

A key element of future studies should be the use of efficacy biomarkers, which enable the objective evaluation of therapeutic outcomes. Such indicators include cytokine profiles (IL-6, TNF-α, IL-10), redox balance markers (GSH/GSSG ratio, SOD, catalase, glutathione peroxidase activity), and oxidative and mitochondrial stress parameters. In recent years, increasing attention has also been given to the analysis of the gut microbiome, whose balance may play an essential role in modulating immune function and response to ozone therapy. The use of advanced omics technologies (metagenomics, metabolomics, proteomics) could provide a more precise understanding of the interactions between ozone and the host microbiological environment, contributing to the development of personalized therapeutic strategies.

In the long term, ozone may be recognized as a potential immune training agent capable of inducing adaptive changes in immune and redox responses, thereby strengthening innate immunity and reducing the incidence of infections. This concept, based on the hormetic effect of low ozone doses, opens up new possibilities for preventing viral and bacterial infections, as well as for supporting immunity under conditions of immunosuppression. However, detailed studies are required to assess the long-term immunological effects, including the durability of the adaptive response, the influence on NK cells, macrophages, and T lymphocytes, and the epigenetic regulation of genes involved in oxidative stress and inflammation.

From a future perspective, ozone therapy may become integrated into the paradigm of personalized medicine and the emerging field of nutrimmunology, which emphasizes comprehensive immune modulation through environmental factors, diet, and the microbiome. Combining ozone therapy with individually tailored antioxidant supplementation (e.g., coenzyme Q10, vitamin C, polyphenols) and dietary interventions could enhance therapeutic efficacy and reduce adverse effects. The development of integrated therapeutic protocols that consider a patient’s genetic and metabolic profile may, in the future, form the foundation for modern, holistic therapeutic strategies utilizing ozone as a modulator of immune and oxidative homeostasis.

Consequently, further research on ozone therapy should aim to develop validated models for assessing clinical efficacy, utilizing molecular biomarkers, biochemical and microbiological parameters, and analyses of long-term immunological outcomes. The integration of ozone therapy with the principles of precision and immunometabolic medicine offers the prospect of its rational use as an innovative tool in the prevention and supportive treatment of immune dysfunctions.

## 7. Limitations

Despite the growing interest in ozone therapy and its potential therapeutic applications, several important limitations must be acknowledged. First, many of the clinical studies assessing the efficacy and safety of medical ozone are characterized by small sample sizes and heterogeneous study designs, which substantially limit the robustness and generalizability of the findings. Moreover, a considerable proportion of available studies are short-term, often lasting only several weeks, which may not sufficiently capture the delayed or cumulative effects of repeated ozone exposure. This is particularly relevant given that some therapeutic protocols recommend long-term or cyclical administration, the consequences of which remain insufficiently documented.

Another significant limitation is the lack of standardization in ozone administration protocols. Variability exists in the applied ozone concentrations, delivery routes (e.g., autohemotherapy, rectal insufflation, topical administration), treatment frequency, and oxygen–ozone ratios. Such inconsistency complicates the comparison of outcomes across studies and hinders the development of clear therapeutic guidelines. In addition, the absence of universally accepted quality standards for ozone-generating equipment may contribute to differences in purity, concentration stability, and reproducibility, further affecting treatment outcomes.

While some studies suggest positive effects of ozone therapy in conditions such as chronic wounds, ischemic disorders, or as an adjunctive antimicrobial agent, the majority of available research compares ozone therapy to placebo or standard supportive care, rather than to established pharmacological treatments. This lack of head-to-head trials significantly restricts conclusions about ozone’s relative efficacy and its potential role as a complementary or alternative therapeutic modality.

Safety concerns must also be considered. Although professionally administered medical ozone is generally regarded as low risk when used according to established protocols, accidental inhalation of ozone is known to cause respiratory irritation, oxidative stress, and potential lung injury. Importantly, isolated case reports describe thrombotic events, air embolism, or adverse neurological reactions following improperly conducted autohemotherapy procedures. The current literature provides limited long-term safety data, and most studies exclude individuals with conditions that may predispose them to complications, such as cardiovascular disease, coagulation disorders, or chronic respiratory pathologies. Consequently, the real-world risk profile of ozone therapy, especially in high-risk populations, remains insufficiently characterized.

Another gap relates to the potential interactions of ozone therapy with other treatments, including anticoagulants, immunomodulatory agents, or oxygen-based therapies. To date, these interactions have not been systematically studied, despite ozone’s well-documented oxidative properties, which could theoretically alter drug metabolism, modify inflammation pathways, or exacerbate underlying disease processes.

Finally, there is a notable discrepancy between the controlled conditions of clinical studies and the unregulated use of ozone therapy in private clinics, where protocols may differ substantially from medically validated approaches. The widespread commercialization of ozone-based treatments, often without adequate monitoring or adherence to medical standards, further complicates the assessment of efficacy and safety. Until rigorous, standardized, long-term randomized controlled trials are conducted, conclusions regarding the therapeutic value of ozone should be considered preliminary.

## 8. Conclusions

This study aimed to provide a detailed analysis of the immunomodulatory mechanisms of ozone action in the context of patients with inborn errors of immunity (IEIs) and secondary immunodeficiencies (SID). The research objective was achieved through a discussion of the hormetic effect, the role of ozone in regulating inflammatory mediators, and the modulation of the immune response at both molecular and cellular levels. The paper also presented the characteristics of local and systemic ozone therapy, its safety profile, degree of standardization, and current legal status in clinical practice.

At low therapeutic concentrations, ozone induces a hormetic effect characterized by the activation of adaptive pathways, particularly the Nrf2/ARE axis, leading to increased expression of antioxidant enzymes such as superoxide dismutase (SOD), catalase (CAT), and glutathione peroxidase (GPx). This activity supports the restoration of redox homeostasis and protects cells against excessive oxidative stress resulting from the overproduction of reactive oxygen species (ROS). Furthermore, ozone exhibits immunomodulatory properties by modifying the cytokine profile, reducing the expression of pro-inflammatory cytokines (IL-1β, IL-6, TNF-α), and stimulating the secretion of anti-inflammatory cytokines (IL-10, TGF-β). At the cellular level, ozone influences the activity of macrophages, regulatory T cells (Treg), and NK cells, thereby enhancing immune function in individuals with recurrent infections and chronic inflammation.

It has been demonstrated that ozone therapy, applied both locally (e.g., ozonated water, ozonated oils) and systemically (e.g., ozone autohemotherapy or rectal insufflation), can serve as an adjunct to conventional treatments, enhancing immune parameters and reducing the frequency of infections. The available data suggest that ozone therapy does not replace standard medical treatments but rather acts as a supportive modality, particularly in patients with chronic inflammation, impaired innate immunity, and increased susceptibility to infections.

The analysis highlights the crucial role of the hormetic effect of ozone in inducing adaptive mechanisms, regulating the expression of antioxidant response genes, and modulating the cytokine profile of immune cells. It also indicates the potential synergistic effects of ozone with other immunomodulatory approaches, including interventions derived from immunonutrition and nutrimmunology, which may represent a promising direction for personalized medicine.

Nevertheless, current research remains limited by the scarcity of long-term, randomized clinical trials and the lack of standardized therapeutic protocols that define optimal doses, administration routes, treatment frequencies, and patient qualification criteria. Moreover, interindividual variability in response to ozone therapy complicates the objective assessment of its efficacy. Therefore, multicenter, prospective clinical studies are warranted to evaluate the safety, effectiveness, and long-term effects of ozone therapy in diverse patient populations. Simultaneously, the development and implementation of international clinical guidelines for ozone therapy are necessary to ensure its standardization, validation, and integration within the framework of evidence-based medicine (EBM).

**Figure 2:**
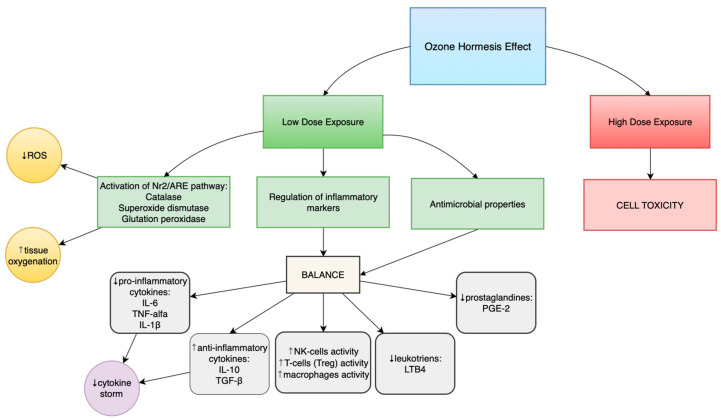
Immunomodulatory properties of ozone. Hormetic effect. ROS—Reactive Oxygen Species, Nr2/ARE pathway—Nuclear factor erythroid 2–related factor 2/Antioxidant Response Element pathway, IL-6—Interleukin 6, TNF-alfa—Tumor Necrosis Factor alpha, IL-1β—Interleukin 1 beta, IL-10—Interleukin 10, TGF-β—Transforming Growth Factor beta, NK-cells—Natural Killer cells, Treg—Regulatory T cells LTB4—Leukotriene B4, PGE-2—Prostaglandin E2.

In conclusion, the interdisciplinary approach adopted in this study—integrating recent advances in molecular biology, immunology, pharmacology, and clinical medicine—has enabled a comprehensive evaluation of ozone therapy. As a potential tool for supporting immunological and redox homeostasis, ozone therapy requires further clinical validation but represents a promising prospect for future strategies in the treatment and prevention of immune-related disorders. Figure 2 summarises ozone’s immunomodulatory properties and hormetic effect.

## Figures and Tables

**Table 1:** Immunomodulatory effects of ozone.

Mechanism of Action	Description of Biological Effect	Main Pathways and Mediators	Clinical/Immunological Effect
Hormetic effect of low ozone doses (10–40 µg/mL)	Induction of mild oxidative stress leading to activation of adaptive cellular mechanisms	Nrf2/ARE pathway, NF-κB regulation, activation of antioxidant enzymes: SOD, catalase, GPx	Enhanced innate immunity, reduced chronic inflammation, restoration of redox homeostasis
Cytokine profile modulation	Reduction in pro-inflammatory cytokines (TNF-α, IL-1β, IL-6) and increase in anti-inflammatory ones (IL-10, TGF-β)	Inhibition of NLRP3 inflammasome, decreased caspase-1 activity	Balanced immune response, prevention of cytokine storm
Effect on macrophages	Increased phagocytic capacity and antioxidant enzyme activity	Activation of Nrf2/ARE, increased SOD, CAT, GPx	Improved pathogen clearance, attenuation of chronic inflammation
Effect on regulatory T cells (Treg)	Increased number and activity of Tregs, induction of FoxP3 expression	Increased IL-10, TGF-β; regulation of microRNAs (miR-17, miR-27, miR-146a)	Suppression of autoimmune reactions, restoration of immune tolerance
Effect on NK cells	Enhanced cytotoxic activity of NK cells	Reduction in TNF-α, increase in IL-10	Strengthened antiviral and antitumor immunity
Regulation of lipid inflammatory mediators	Inhibition of excessive prostaglandin (PGE2) and leukotriene (LTB4) production	Inhibition of COX and 5-LOX	Reduced chronic inflammation, stabilization of immune response
Restoration of redox homeostasis	Reduction in ROS, improvement of GSH/GSSG balance, activation of antioxidant enzymes	Nrf2/ARE, SIRT1–Nrf2/HO-1	Protection against oxidative damage, immune cell regeneration
Effect on gut microbiome	Modulation of microbiota composition, increase in Lactobacillaceae and Adlercreutzia	Activation of SIRT1–Nrf2/HO-1 pathway	Improved intestinal barrier integrity and mucosal immunity

**Table 2:** Medical applications of ozone.

Type of Application	Form of Therapy	Scope/Clinical Indications	Effects Confirmed by Studies	Dose Range/Safety
Systemic ozone therapy (autohemotherapy)	Ozonation of blood followed by reinfusion	PI, SI, HIV, COVID-19, autoimmune diseases, chronic inflammation	Increase in CD4/CD8 lymphocytes, improved cytokine balance, decreased CRP and IL-6	10–40 µg/mL—hormetic effect, no adverse reactions
Ozonated water therapy (local/rectal)	Ozonated water, enemas or irrigations	Gut barrier disorders, intestinal infections, mucosal inflammation	Activation of SIRT1–Nrf2/HO-1, improved microbiome, epithelial regeneration	10–30 µg/mL, high safety profile
Topical applications (dermatology, dentistry, surgery)	Ozonated water or oil	Chronic wounds, ulcers, oral infections, postoperative complications	Antimicrobial effect, accelerated healing, improved tissue oxygenation	60–100 µg/mL (topical)—disinfectant action
Supportive ozone therapy in rheumatology and orthopedics	Autohemotherapy, insufflation, intra-articular injections	Degenerative and inflammatory joint diseases, musculoskeletal pain	Reduced IL-6 levels, improved microcirculation, pain relief	10–40 µg/mL, effects last up to 6 months
Adjunctive therapy in oncology and immunosuppression	Autohemotherapy or insufflation	Immunodeficiency after chemotherapy, secondary infections	Reduced oxidative stress, improved phagocyte and NK cell functions	Individually adjusted, under clinical supervision
Synergy with immunonutrition	Ozone + micronutrients (Se, Zn, vit. C, Q10)	Immune dysfunction, chronic oxidative stress	Synergistic antioxidant and immunomodulatory effects	According to WFOT/ISCO3 guidelines

## Data Availability

No new data were created or analyzed in this study. Data sharing is not applicable to this article.
